# Diversity and Distribution Patterns of Amphibians in the Huangshan Mountain Region: The Roles of Climate and Human Activities

**DOI:** 10.3390/ani15070938

**Published:** 2025-03-25

**Authors:** Fei Hong, Dapeng Pang, Xiaojia Lin, Weixin Huang, Jie Fang, Wenbo Li

**Affiliations:** 1 School of Life Sciences, Anhui University, No. 111, Jiulong Road, Hefei 230601, China; ahufeihong@126.com (F.H.); pia@163.com (D.P.); 15217599516@163.com (W.H.); 2 International Collaborative Research Center for Huangshan Biodiversity and Tibetan Macaque Behavioral Ecology, No. 111, Jiulong Road, Hefei 230601, China; 3 Technology Center of Hangzhou Customs District, Hangzhou 310016, China; lxj@zaiq.org.cn; 4 School of Resources and Environmental Engineering, Anhui University, No. 111, Jiulong Road, Hefei 230601, China

**Keywords:** climate change, human activities, amphibians, species richness, biodiversity

## Abstract

Global climate change and human activities are significant threats to biodiversity, contributing to the endangerment of approximately 41% of amphibian species worldwide. In this study, we applied field survey methods, the MaxEnt model, and integrated climate and human activity data to predict potential changes in the diversity and distribution of amphibian species in Huangshan Mountain, China. In this study, we have found 23 amphibian species, belonging to two orders, eight families, and 18 genera. The MaxEnt models showed that the distance from farmland (contributing 26.2%), shrubs (15.6%), and waterbodies (10.6%), as well as the NDVI (Normalized Difference Vegetation Index) (10.1%), significantly influence species distribution and diversity, suggesting that amphibian species prefer habitats with lower levels of human disturbance. Our models also showed that Bio3 (isothermal) (8.9%) and Bio8 (mean temperature of wettest quarter) (8.6%) have a significant impact on the species distribution and diversity, suggesting that amphibians are influenced by temperature and humidity. Our field survey showed that seasonal variation in amphibian diversity revealed significant correlations between climatic factors. Specifically, amphibian species diversity was positively correlated with wind speed, soil moisture, and rainfall (*p* < 0.05), while amphibian abundance was significantly linked to soil temperature, soil moisture, and rainfall (*p* < 0.05). These findings underscore the critical role of both climatic conditions and habitat structure in shaping amphibian populations and their distribution in Huangshan Mountain. Therefore, local management authorities should continue to monitor the marginal areas of the region, taking into account key human disturbances and climatic factors that favor the formation of amphibian diversity hotspots. Protective buffer zones should be established to provide effective refuges for amphibians.

## Simple Summary

In this study, we employed field surveys, the MaxEnt model, and integrated climate and human activity data to project potential changes in the distribution range and diversity of amphibian species in Mount Huangshan, China. The results revealed that both Shannon and Simpson indices were lowest in March and peaked in May. In addition to rainfall, soil temperature, humidity, and wind speed were identified as factors influencing amphibian diversity in mountainous regions, expanding our understanding beyond conventional precipitation effects. Furthermore, MaxEnt modeling of 18 out of the 23 amphibian species indicated their concentration in mid- to low-altitude areas around Huangshan’s main peak, forming hotspots with notable edge effects. Our modeling also highlighted agricultural habitats as primary factors influencing the distribution of amphibian hotspots in local areas, suggesting that appropriate human interventions could enhance species diversity. Therefore, it is crucial to focus on edge areas, considering climatic conditions and human activities, and establish protective buffer zones to effectively safeguard amphibian populations.

## 1. Introduction

Anthropogenically driven environmental degradation poses severe threats to global biodiversity, with Earth potentially entering its sixth mass extinction event [1,2]. Currently, over 46,300 species worldwide face extinction risks, including more than 500 terrestrial vertebrate species that have been declared or presumed extinct within the past five centuries [3]. Of particular concern are amphibians, which represent one of the most imperiled vertebrate groups—41% of extant species are categorized as threatened with extinction [4,5]. China, harboring 695 amphibian species, stands as one of the world’s most biodiverse nations for this taxon [6]. However, under current anthropogenic and climatic pressures, Chinese amphibians are projected to lose an average of 20% of their extant habitats [7]. Recent studies further underscore alarming population declines in critically endangered (CR) species such as *Andrias davidianus*, emphasizing the urgency of targeted conservation interventions [8].

Significant changes in the natural environment, driven by human activities and climate change, have forced most species into fragmented habitats, a major factor contributing to ongoing threats to amphibian populations [9,10]. Amphibians, due to their restrictive physiological requirements and low mobility, cannot track shifting environments like species with strong dispersal abilities [11]. When environmental conditions within their range can no longer support a stable population, amphibians often experience population declines and range contractions [12,13]. For instance, over the past 40 years, amphibians on the Tibetan Plateau have abandoned their southwestern range and shifted toward the central region, seeking more suitable temperatures and solar radiation [13]. Mountainous climates, as a unique aspect of the ongoing global climate change, may be altering amphibians’ survival patterns. Global climate change has already affected seasonal temperature cycles, which in turn influence the survival patterns of amphibians [14]. Some studies, however, suggest that the steep terrain of mountainous regions harbors diverse microclimates that may differ significantly from regional climate patterns [15,16,17]. These microclimates, shaped by terrain, canopy cover, and the decoupling of local and regional climate patterns, may mitigate global warming effects [16]. Global efforts have been made to address the exacerbated loss of amphibians due to climate change, identify biodiversity hotspots, and propose conservation strategies. However, most studies rely on long-term or decadal climate models to analyze species at the global scale [3,5]. Few studies focus on the effects of climate change on amphibian diversity at shorter timescales and smaller regional scales, especially in mountainous areas with unique climatic conditions.

Similarly, some studies show that 85.7% of the world’ s mountainous areas are affected by human activities, contributing to increasing biodiversity threats and disturbances [18]. Human activities, such as deforestation, road construction, and tourism expansion, significantly impact ecosystem biodiversity [19,20]. These activities fragment and destroy habitats, forcing species to shift ranges or face extinction risk [21,22]. The most common recorded threats to all threatened amphibians are habitat loss and degradation [4]. The top three threats are agriculture (affecting 77% of species), timber and plant harvesting (53%), and infrastructure development (40%) [23]. These threats may be exacerbated in mountainous regions. In mountain forest ecosystems, amphibians may face more severe threats despite their key ecological roles [17,24]. In some forests, amphibians contribute more to vertebrate biomass than birds and mammals combined [25,26]. However, mountain forest ecosystems have experienced some of the most significant land-use changes in recent years. In China, for example, some mountain areas, heavily reliant on timber, have engaged in extensive deforestation over the past few decades [27]. In response to the growing emphasis on ecological conservation and sustainable development, China launched the Natural Forest Protection Program (NFPP) in 1999 to enhance forest cover [27]. However, the dominance of monoculture plantations has disrupted biodiversity patterns [28]. This ecological shift has also threatened mountain amphibians’ habitat ranges, leading to regional extinctions of some species [17]. Meanwhile, the growing demand for tourism in recent years has led to the development of large areas of pristine mountain forests into tourist attractions [17]. The expansion of tourism infrastructure has further stressed mountain amphibians’ living conditions. Although much attention has focused on quantifying past species losses, urgent consideration is also needed to protect existing biodiversity [29,30].

Huangshan Mountain, a UNESCO World Heritage site and one of China’ s first national key scenic spots, is located within the Huangshan-Huaiyu Mountains National Biodiversity Conservation Priority Area [31,32]. It is one of China’s most biodiverse regions, located at approximately 30° N latitude, and represents a typical mountain forest system [33]. Previous studies have expanded our understanding of amphibian diversity in Mt. Huangshan [34,35,36]. However, the total number of amphibian species in the region is still unknown, and much of the area remains unexplored. For example, there is a lack of data on the species diversity, distribution patterns, and population dynamics of amphibians in this area. Between 1990 and 2004, the annual number of visitors increased dramatically from 669,800 in 1990 to 1.60 million in 2004, nearly a 2.5-fold increase [31]. In October 2019, the number of domestic and international tourists surpassed 3 million for the first time. By August 2023, the number had exceeded 3 million, two months earlier than in 2019. By the end of the year, the total number of visitors reached 4.57 million, setting a new historical record (https://www.ah.chinanews.com.cn/news/2024/0101/321804.shtml, accessed on 10 November 2024). In recent years, the growth of tourism has led to the continuous construction and renovation of roads, scenic spots, and buildings. Moreover, the economic benefits from tourism have increased local farmers’ incomes, reducing their reliance on farmland. As a result, most farmland has been converted into forest land [31]. In the context of these land-use changes, the composition, distribution, and habitat preferences of amphibian populations remain unclear. Additionally, Huangshan’s well-established ecological monitoring infrastructure, including meteorological stations along a full altitudinal gradient, offers a valuable opportunity to study how local climatic factors in mountainous regions influence amphibian diversity.

Given the urgent need to understand the impacts of climate change and human disturbance on amphibian diversity in mountainous regions, this study focuses on long-term research in Mt. Huangshan. First, we use the MaxEnt model to simulate and predict amphibian diversity distribution. In addition to 19 commonly used climatic factors, we include human disturbance factors, such as the distance to farmland, roads, and water sources, to identify key climate and human activity drivers of amphibian diversity. Second, we aim to examine the species composition and vertical distribution patterns of amphibians in the Huangshan area. Third, we analyze seasonal variation in amphibian diversity and collect local climate data to identify the key climatic drivers of these changes. Finally, we combine the model results, considering both human disturbance and climate factors, to discuss the underlying causes of amphibian diversity distribution and variation in the region. This study provides baseline data for amphibian habitat protection in the region and offers localized, precise insights into how species are responding to large-scale human activities and climate change.

## 2. Materials and Methods

### 2.1. Study Area

**Figure 1:**
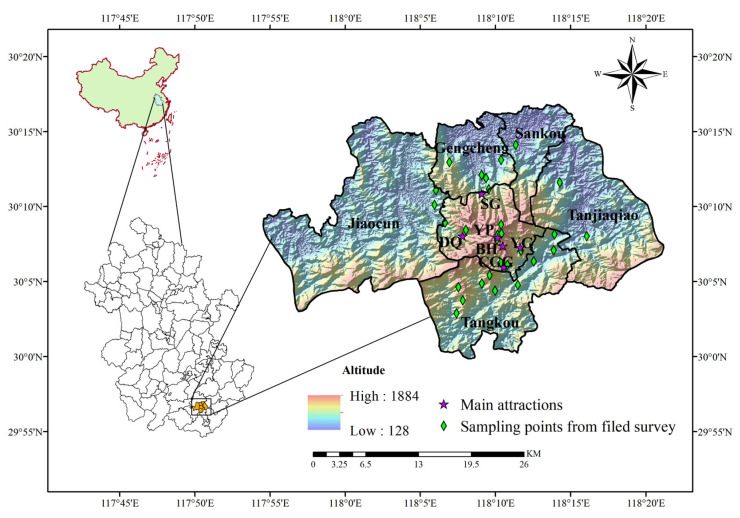
The map of the study area and sampling points in Mt. Huangshan.

This study was conducted in Mt. Huangshan, located in the southern part of Anhui Province, China. The main area of Huangshan Scenic Area covers 160.6 km^2^ (30°11′ N, 118°10′ E). The highest peak reaches an elevation of 1884 m [33]. The main attractions in the area include Ci Guangge (CCG), Yun Gu (YG), Song Gu (SG), Diao Qiao (DQ), Yu Ping (YP), and Bei Hai (BH). Surrounding the main attractions are five townships: Tangkou, Tanjiaqiao, Gantang, Gengcun, and Jiaocun. This study covers the main area of Huangshan Scenic Area and the five adjacent townships (Figure 1).

### 2.2. Prediction of Amphibian Richness Based on the MaxEnt Model

The MaxEnt model, based on maximum entropy theory, is one of the most widely used ecological niche models for simulating species distribution patterns [37]. It uses species distribution data and environmental variables to predict potential distribution areas of species [37]. MaxEnt exhibits superior performance in forecasting accuracy, especially in the case of lacking species distribution data [38,39,40]. In this study, we used the MaxEnt model to predict the current distribution of amphibians in Mt. Huangshan and subsequently mapped the potential species richness of amphibians.

(a) Occurrence data

In order to construct a species distribution model (SDM) for amphibians in the Mt. Huangshan biodiversity hotspot, amphibian occurrence records were compiled through an extensive survey of Mt. Huangshan (see Section 2.3 Survey Methods). These records collectively characterize the biodiversity hotspot.

**Figure 2:**
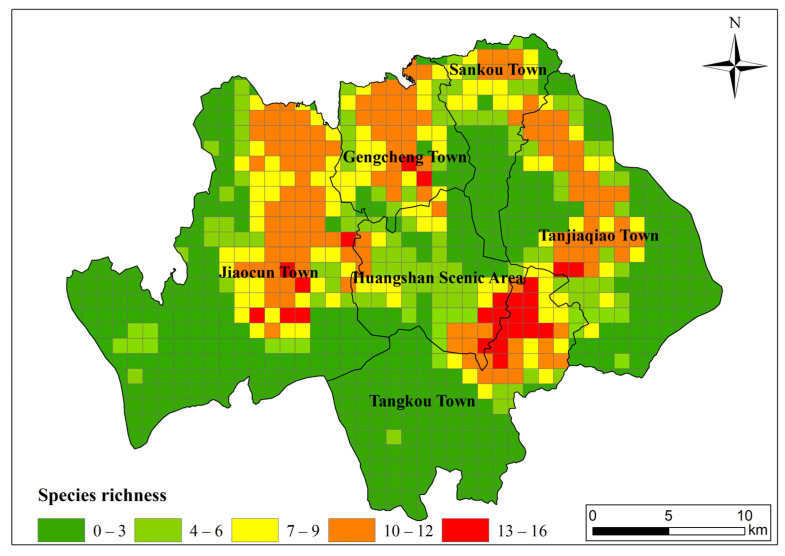
Potential species richness of amphibian species.

However, during our survey, fewer than 10 occurrence sites were identified for five amphibian species, which were excluded from our analyses to ensure accurate model predictions for the distribution of other species. Thus, a total of 18 amphibian species and 381 occurrence points (Figure 2) were analyzed in this study, and random points were generated from the raster map of China using ArcGIS 10.8 (ESRI, Redlands, CA, USA).

(b) Environmental predictors

We considered 26 environmental predictors that included 19 bioclimatic variables, vegetation characteristics, terrain factors, and human disturbance factors (Table 1). Current (1950–2000 average) and future bioclimatic variables were obtained from WorldClim (http://www.worldclim.org, accessed on 25 June 2024) with a resolution of 30 s. Topographic factors, including altitude, were extracted using spatial analysis tools from the 90 m resolution SRTM v4 digital elevation model (http://srtm.csi.cgiar.org, accessed on 28 June 2024). The normalized difference vegetation index (NDVI), and land use data were obtained from the Resource and Environmental Science Data Center of the Chinese Academy of Sciences (https://www.resdc.cn/Default.aspx, accessed on 28 June 2024). Given the life history characteristics of amphibians, which are thought to require complex wetlands, the distance to water bodies was obtained from the National Earth System Science Data Center (www.geodata.cn, accessed on 28 June 2024). Additionally, considering the human disturbance in Mt. Huangshan, the distance to farmland and distance to roads were obtained from the National Basic Geographic Information System (http://www.ngcc.cn, accessed on 28 June 2024) and calculated using Euclidean distances.

All environmental raster data were resampled to a 1 km resolution and projected onto the WGS 1984 UTM Zone 49 N coordinate system. Multicollinearity among the variables was assessed using SDMtoolbox [38]. For pairs of highly correlated variables (Pearson correlation coefficient |r| ≥ 0.8), those with biological relevance were retained, as suggested by [39] (see Table 2 for further details).

### 2.3. Survey Methods

Sampling points were established based on amphibian habitat preferences to ensure survey feasibility and to cover diverse habitats, including montane forests, mountain streams, dry fields, fire prevention ponds in high-altitude areas, and rural ponds. A sampling line was set for each point, with each spline measuring 300 m in length and 5 m in width. Finally, a total of thirty splines was established, covering the range of amphibian habitats in the study area (Figure 1) [41].

Species surveys were conducted using the line sampling method. The surveys were conducted in 2023. We reviewed previous literature on species recorded in the region and selected a survey period from March to September, focusing on the middle of each month to minimize error and align with the local monthly climate patterns. Two researchers were involved: one recorded species data along the sampling line, and the other collected specimens and took photographs. Species were identified using a combination of visual observation (specimen collection or photographs) and molecular sequencing. Species identification in the field followed Chen [42], with one male and one female specimen preserved for each species. For certain protected species, tissue samples (e.g., toes) were collected for molecular identification. The collected specimens were fixed in 10% formalin, rinsed, and stored in 75% ethanol. For species that are difficult to identify or potentially cryptic, liver or toe tissue was stored in 95% ethanol at −80 °C for DNA barcoding.

To examine the relationship between amphibian diversity and climatic factors in the study area, climate data were collected from seven ecological monitoring stations. This included five primary monitoring stations located at altitudes of 900–1800 m in Song Gu, Diao Qiao, Ci Guang Ge, Yun Gu Si, and Tian Hai, as well as two secondary monitoring stations at altitudes of 200–600 m in the Fuxi Tourist Area and Tangquan Hotel. The study analyzed eight environmental variables: wind speed (F), atmospheric temperature (DT), atmospheric humidity (RH), light intensity (LUX), soil temperature (TW), soil humidity (TS), soil pH (TPH), and rainfall (JY).

### 2.4. Data Analysis

#### 2.4.1. Model Approach

We used MaxEnt version 3.3.4 to predict the potential distribution of 18 species [37]. MaxEnt estimates species’ distributions by calculating the most uniform distribution (i.e., maximum entropy), subject to the constraint that the expected value of each environmental variable matches the empirical average of the locality data [43]. Importantly, MaxEnt generates a probability distribution for habitat suitability (based on an index) across the study area [37], enabling comparisons of suitability estimates among regions. MaxEnt can also estimate each variable’s contribution to the ENM using a jack-knife analysis of the gain. Gain is a unitless statistic that evaluates how well the predicted distribution fits the occurrence data compared to a uniform distribution [37].

#### 2.4.2. Diversity Analysis

For amphibian species diversity, five different measures of species diversity were used to analyze the amphibian data over the seven months.

(a) Dominance Index

The dominance index (Berger–Parker Index) measures the degree of dominance of a species in a community, typically the proportion of that species in the community. It can be used as a measure of dominant species. The calculation method is as follows:

I = ni/N

where I represents the dominance index, ni is the number of individuals of species i, and N is the total number of individuals.

(b) Shannon–Wiener Index

The Shannon–Wiener index is used to investigate the diversity of species within a local habitat (α-diversity). The calculation method is as follows:

H = −∑(P_i_)(lnP_i_)

where P_i_ is the proportion of individuals of species i. When the number of species is less than 2, H is 0 [44].

(c) Simpson Index

This study used the Gini–Simpson index (GS) to calculate species richness, with the following formula:

$$\mathrm{GS}=1-\sum_{i=i}^{s}{P_{i}}^{2}$$

where S is the community species richness index, i.e., the total number of species; P_i_ is the relative abundance of species i [45].

(d) Evenness Index [46]

Pielou’s evenness index was used to determine the evenness of amphibian communities:

$$J=\frac{H\prime }{H_{\max}}(H\;\max=\ln S)$$

(e) Chao1 Index

The Chao1 index is used in ecology as a measure of species richness. A higher value indicates a richer community.

The classic formula for the Chao1 index is as follows:

$$\mathrm{Chao}1=S+\frac{{F_{1}}^{2}}{2F_{2}}$$

S: community species richness index; F_1_: the number of species containing only one individual; and F_2_: the number of species containing only two individuals [47].

#### 2.4.3. Correlation Analysis

Environmental data from seven observation stations in 2023 were used, and daily averages from March to July were standardized to derive monthly environmental averages. This study correlated the processed environmental data with monthly amphibian species data using a Spearman correlation matrix to assess the relationships between species count, richness, Shannon–Wiener diversity index, Simpson dominance index, and Pielou evenness index with environmental factors. R (using the psych package) was employed to compute monthly data for amphibians and environmental variables [48]. Spearman correlation coefficients were used, with *p*-values adjusted using the Benjamini method by default [48]. The ggplot2 package was used to create heatmaps displaying correlation coefficients between amphibians and environmental variables, with colors indicating correlation strength [49].

## 3. Results

### 3.1. Species Richness

MaxEnt showed great predictive performance for all amphibians, with high values for AUC (>0.8). The AUC scores ranged from 0.73 for *Odorrana tormota* to 0.92 for *Hyla chinensis*, and the average was 0.86 ± 0.04. The mean AUC value demonstrates the model’s strong predictive performance.

According to the jack-knife analyses of variable importance, the distance to farmland most influenced all species (12 of 18 species). Based on each percentage contribution estimate, the distance to farmland provided the most information to all models (26.2%), followed by elevation (15.6%) and NDVI (10.6%), respectively (Table 2). Detailed data are presented in Table S1.

This study explores the geographic distribution pattern of amphibian species richness based on a 1 km × 1 km grid. Spatial distribution modeling of species richness indicates that hotspots (areas of high species richness > 12) are primarily concentrated in the middle to lower elevations around the main peaks of Mt. Huangshan. Most hotspots are located around the periphery of the scenic area and in the central parts of townships (Figure 2). The elevation range from 200 to 1800 m is divided into three levels: low (200–700 m), middle (700–1200 m), and high (above 1200 m). It was found that the proportion of high species richness grids within the 200–600 m elevation range is the highest (80.8%), significantly surpassing the middle (19.2%).

### 3.2. Species Diversity and Altitude Pattern

A total of 1982 adult amphibians were recorded, including 23 amphibian species, belonging to two orders, eight families, and 18 genera (Table S2). The dominant species (I > 0.1) were *Amolops wuyiensis* and *Fejervarya multistriata*, with six rare species (I < 0.01): *Cynops orientalis*, *Rana catesbeiana*, *Hyla sanchiangensis*, *Rana zhenhaiensis*, *Limnonectes fujianensis*, and *Zhangixalus dennysi* (Table S2). Endangered status assessments for the 23 amphibian species in the study area were conducted based on the IUCN (2024) Red List criteria [4]. The results show that, among the 23 amphibian species in Mt. Huangshan, two species were not assessed (10%), 13 were classified as Least Concern (62%), three as Near Threatened (14%), two as Vulnerable (9%), and one as Critically Endangered (5%).

**Figure 3:**
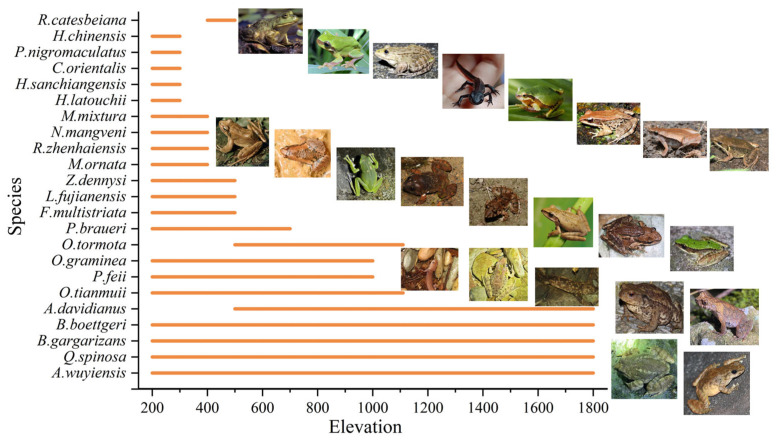
Altitude distribution patterns.

Amphibians are distributed across various altitudinal transects, with species richness decreasing along the elevational gradient. The 200–600 m altitude range includes all amphibian species found in Mt. Huangshan. The altitudinal zone that divides species diversity is at 1200 m, where only five amphibian species are found above this elevation, accounting for 22% of the total species. The remaining species occur within the 200–600 m range, indicating a broad ecological niche (Figure 3).

### 3.3. The Seasonal Variation in Amphibians’ Response to Climatic Factors

**Figure 4:**
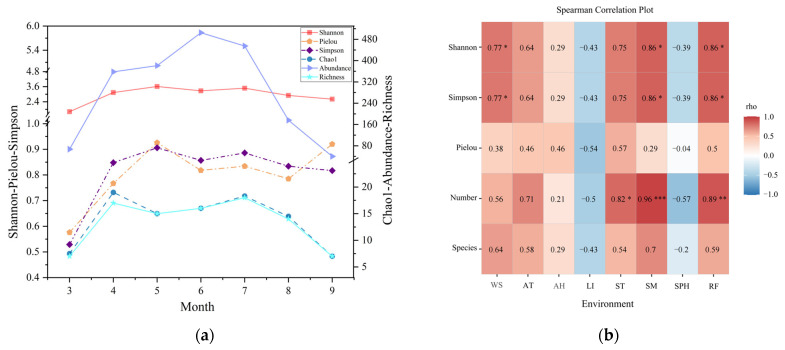
Amphibian diversity index across different months (**a**) and correlation with environmental factors (**b**). (“*” indicates *p* < 0.05, “**” indicates *p* < 0.01, and “***” indicates *p* < 0.001. WS: wind speed, AT: atmospheric temperature, AH: atmospheric humidity, LI: light intensity, ST: soil temperature, SM: soil moisture, SPH: soil pH, and RF: rainfall).

We found the richness and abundance of mountain amphibian species vary significantly by month, with the highest richness of species in July (18 species) and the lowest in March and September (7 species). The highest abundance was recorded in June (505 individuals), while the lowest was recorded in September (40 individuals). Both the Shannon and Simpson indices are lowest in March and highest in May. The Chao1 index results indicate that the highest number of amphibian species occurred in April, and the lowest in September (Figure 4a, Table S3).

The correlation matrix indicates significant positive correlations (*p* < 0.05) between wind speed, soil moisture, and rainfall with the Shannon–Wiener diversity index and Simpson index for amphibians. Additionally, the amphibian species count shows significant positive correlations (*p* < 0.05) with soil temperature, soil moisture, and rainfall, with soil moisture exhibiting a particularly strong correlation (*p* < 0.001) with species count (Figure 4b).

## 4. Discussion

This study documents 23 amphibian species in the Huangshan Scenic Area, representing 46% of the known amphibian diversity in Anhui Province [50]. Previous surveys identified 17 species within the core protected zones; however, their limited spatial scope (restricted to Tangkou Town and the interior of the scenic area) and exclusive reliance on morphological identification—which is now considered insufficient for detecting cryptic diversity in amphibians—emphasize the need for our integrative approach [34,35,51]. By combining systematic transect surveys, DNA barcoding, and morphological identification, we not only updated the regional species checklist but also uncovered new distribution patterns. Specifically, all recorded amphibian species were found within the 200–600 m altitude range, with biodiversity hotspots concentrated in anthropogenic edge habitats, notably at farmland–shrubland ecotones. This integrated framework provides critical insights for balancing conservation priorities in mountainous landscapes that are rapidly transforming due to tourism.

Human activities and climate change have caused extensive habitat fragmentation, posing significant challenges for biodiversity conservation [3,10,14]. While studies suggest that amphibians tend to shift toward higher elevations in response to global warming, our findings in Huangshan reveal that these species predominantly inhabit lower-elevation peripheral zones [52]. Our results suggest that this counterintuitive distribution arises from a synergistic interaction between favorable microclimatic conditions (temperature and humidity) and human-altered landscapes (farmland–shrubland). This finding underscores the critical role of temperature and humidity—not only of the atmosphere but also at the soil level—in shaping amphibian distributions [53,54].

Previous research has largely focused on atmospheric conditions, but our study suggests that microhabitat parameters should be a focal point for future conservation efforts [54,55]. It is worth noting that soil temperature and moisture are key indicators of amphibian habitat suitability, particularly for species that brumate underground or rely on specific daytime refuges [55]. As ectothermic organisms, prolonged exposure to sublethal environmental conditions may induce behavioral modifications or mortality in amphibians [56,57]. The maintenance of their cutaneous respiration necessitates terrestrial substrates with moderate humidity and robust vegetative coverage, which collectively preserve integumentary moisture [56,58]. Contemporary landscape alterations, particularly urbanization-induced habitat fragmentation and deforestation-generated xeric terrains, have substantially degraded these critical thermoregulatory and hydric buffering capacities [56]. This environmental degradation constitutes a principal mechanistic driver underlying amphibian population declines [56]. In contrast, protected mountainous ecosystems demonstrate preserved microhabitat provisioning capabilities, with our findings revealing these refugia predominantly cluster in mid-to-low elevation zones along montane peripheries. This elevational distribution aligns with the established mid-domain peak pattern of amphibian diversity and its subsequent attenuation with increasing altitude, as documented in prior biogeographic studies [59,60]. In the case of Mount Huangshan, our elevation distribution analysis shows that the 200–500 m range supports the full spectrum of amphibian species, whereas only five species occur above 1,200 m. This pattern is consistent with observed decreases in both air and soil temperatures—with a decline of approximately 0.6 °C per 100 m in elevation—and associated reductions in humidity [61,62].

We also examined the impact of human activities on amphibian diversity. Our findings indicate that amphibian hotspots are predominantly located near farmlands and shrublands. Over the past two decades, rapid tourism development in Huangshan has brought significant economic benefits to local communities, thereby reducing reliance on agriculture [31,63]. As a result, many agricultural lands are now either seasonally cultivated or have been abandoned, leading to secondary forest succession and the expansion of shrublands [31,32]. This moderate disturbance appears to create favorable conditions for amphibian diversity, supporting the intermediate disturbance hypothesis. Interestingly, shrubland habitats themselves show a positive correlation with amphibian diversity. This is in line with other studies that demonstrate increased shrub density can bolster amphibian populations, regardless of local drought conditions [41]. The regional vegetation mosaic of Huangshan—comprising evergreen broadleaf forests (600–1000 m), deciduous broadleaf forests (1000–1500 m), and a dwarf forest zone dominated by *Pinus taiwanensis* at 1500–1700 m—illustrates how human activities have transformed the landscape [33]. In low-elevation areas (<600 m), continuous evergreen broadleaf forests have been fragmented into a patchwork of farmland, grassland, secondary forests, and plantations, leading to the expansion of shrublands [33]. This habitat diversification appears to contribute to the formation of amphibian diversity hotspots.

Finally, the observation that peripheral areas may harbor greater biodiversity than core protected zones is not unique to amphibians. Similar patterns have been reported in studies on mammals in the Huangshan region, where non-protected areas surrounding the scenic core also exhibit higher species diversity [32]. Based on these findings, we recommend that local conservation authorities extend protection measures to these peripheral areas to ensure more effective biodiversity preservation.

## 5. Conclusions

Through detailed surveys of amphibian species in the Huangshan Mountain area, this study elucidated the species composition and dynamics of the region. By integrating climate and human disturbance data, we identified key factors shaping the distribution of amphibian diversity. The study reached the following three conclusions: (1) Huangshan harbors a rich amphibian fauna, with 23 species recorded. (2) Amphibian hotspots are primarily concentrated along the edges of the core scenic area. We recommend the establishment of a buffer zone around the core area as an effective strategy for biodiversity conservation. (3) Soil temperature and humidity, as well as the presence of farmland and shrubland habitats, are significant factors influencing the formation of amphibian hotspots.

While this study is conducted at a local scale, it emphasizes the interplay between mountain forest systems—characterized by significant human activity and unique climatic conditions—local climate factors, and large-scale climate data, alongside changes in amphibian diversity over a restricted range. We suggest that similar studies in other mountain scenic areas, analyzing the key factors influencing amphibian diversity through multiple climate variables and human disturbances, would provide valuable insights. Identifying common patterns across regions is a logical next step that can improve the management and protection of amphibian populations.

## Figures and Tables

**Table 1:** Environmental variable codes and descriptions.

Codes	Environmental Predictors
Bio1	Annual Mean Temperature
Bio2	Mean Diurnal Range
Bio3	Isothermal
Bio4	Temperature Seasonality
Bio5	Max Temperature of Warmest Month
Bio6	Min Temperature of Coldest Month
Bio7	Temperature Annual Range
Bio8	Mean Temperature of Wettest Quarter
Bio9	Mean Temperature of Driest Quarter
Bio10	Mean Temperature of Warmest Quarter
Bio11	Mean Temperature of Coldest Quarter
Bio12	Annual Precipitation
Bio13	Precipitation of Wettest Month
Bio14	Precipitation of Driest Month
Bio15	Precipitation of Seasonality, Coefficient of Variation
Bio16	Precipitation of Wettest Quarter
Bio17	Precipitation of Driest Quarter
Bio18	Precipitation of Warmest Quarter
Bio19	Precipitation of Coldest Quarter
Elev	Elevation
Waterbody_dis	Distance to Waterbody
NDVI	Normalized Difference Vegetation Index
Shrub	Shrub Distribution
Forests	Forests Distribution
Road_dis	Distance to Road
Farmland_dis	Distance to Farmland

**Table 2:** Percentage contribution and permutation importance of environmental variables to the model.

Variables	Percentage Contribution	Permutation Importance
dis_farmland	26.2%	29.16%
dis_shrub	15.6%	11.16%
dis_waterbody	10.6%	11.14%
ndvi	10.1%	10.61%
bio3	8.9%	10.59%
bio8	8.6%	8.94%
Elev	8.4%	7.96%
bio15	6.5%	6.80%
dis_forests	5.1%	3.63%

## Data Availability

All data are available in the open figshare repository, and the link to the data is https://doi.org/10.6084/m9.figshare.26335282 (accessed on 19 July 2024).

## Supplementary Materials

The following supporting information can be downloaded at: https://www.mdpi.com/article/10.3390/ani15070938/s1, Table S1. Percentage contribution and permutation importance of environmental variables to the model; Table S2. List of Amphibian Survey Results in the Mt. Huangshan; Table S3. The Mt. Huangshan Amphibian Diversity Index Across Different Months.
