# Supplementary material for: Diversity and Distribution Patterns of Amphibians in the Huangshan Mountain Region: The Roles of Climate and Human Activities
Source: Animals (Basel). 2025 Mar 25;15(7):938. doi: 10.3390/ani15070938 (PMC11988013; doi:10.3390/ani15070938)
Supplement: Supplementary file 1 [file animals-15-00938-s001.zip › animals-3514368-supplementary.docx]

Supplementary Materials

**Table S1.** Percentage contribution and permutation importance of environmental variables to the model.

|  | **AUC** | **bio15** | | **bio3** | | **bio8** | | **dis_farmland** | | **dis_forests** | | **dis_shrub** | | **dis_waterbody** | | **elev** | | **ndvi** | |
| --- | --- | --- | --- | --- | --- | --- | --- | --- | --- | --- | --- | --- | --- | --- | --- | --- | --- | --- | --- |
| **Species** |  | **PI** | **PC** | **PI** | **PC** | **PI** | **PC** | **PI** | **PC** | **PI** | **PC** | **PI** | **PC** | **PI** | **PC** | **PI** | **PC** | **PI** | **PC** |
| 1. *Boulenophrys boettgeri* | 0.892±0.024 | 3.7 | 4.10% | 21.5 | 15.40% | 7.3 | 15.50% | 12.5 | 7.20% | 0 | 0.20% | 16.9 | 23.70% | 19.2 | 17.70% | 12.9 | 11.20% | 6 | 5.00% |
| 1. *Bufo gargarizans* | 0.91±0.027 | 5.7 | 2.60% | 22.3 | 21.40% | 2.9 | 6.90% | 11.8 | 4.80% | 0 | 1.60% | 33.9 | 30.10% | 9.8 | 14.40% | 6.3 | 10.90% | 7.1 | 7.30% |
| 1. *Fejervarya multistriata* | 0.784±0.052 | 1 | 0.50% | 7.1 | 3.50% | 2.2 | 0.10% | 26.5 | 23.20% | 3.3 | 6.70% | 18.8 | 5.80% | 0.6 | 0.50% | 16.1 | 12.90% | 24.3 | 46.90% |
| 1. *Hyla chinensis* | 0.922±0.03 | 0 | 0.00% | 1.1 | 0.60% | 0 | 0.00% | 88 | 83.60% | 0.7 | 1.00% | 3.2 | 4.30% | 0.1 | 1.60% | 1.9 | 0.80% | 5 | 8.20% |
| 1. *Hylarana latouchii* | 0.830±0.055 | 29.6 | 6.10% | 8.6 | 4.60% | 22.8 | 17.50% | 33.6 | 6.80% | 0.4 | 51.50% | 0.1 | 1.40% | 4.8 | 8.30% | 0 | 0.50% | 0 | 3.40% |
| 1. *Microhyla mixtura* | 0.905±0.027 | 6.6 | 7.10% | 19.4 | 10.80% | 7.5 | 5.10% | 27.7 | 29.30% | 0.3 | 2.60% | 6.3 | 7.00% | 4.8 | 1.10% | 11.9 | 22.90% | 15.6 | 14.10% |
| 1. *Microhyla ornata* | 0.858±0.039 | 1.6 | 3.60% | 6.8 | 17.90% | 4.6 | 9.20% | 13.5 | 9.80% | 8.8 | 5.70% | 14.3 | 20.40% | 35.1 | 18.80% | 13.6 | 11.10% | 1.7 | 3.40% |
| 1. *Nidirana mangveni* | 0.902±0.036 | 22.4 | 18.40% | 4.1 | 3.00% | 29.1 | 15.70% | 25.5 | 21.50% | 0 | 8.10% | 0 | 0.20% | 1.3 | 8.70% | 5.8 | 11.70% | 11.8 | 12.60% |
| 1. *Odorrana graminea* | 0.838±0.07 | 1.6 | 2.60% | 10.4 | 13.00% | 1.6 | 13.80% | 17.4 | 19.70% | 3.6 | 1.30% | 5.5 | 13.70% | 39.1 | 11.50% | 20.7 | 22.40% | 0.2 | 2.00% |
| 1. *Odorrana tianmuii* | 0.895±0.018 | 1 | 0.80% | 5.4 | 3.40% | 18.3 | 7.40% | 54.7 | 56.30% | 1.2 | 5.70% | 0.3 | 0.00% | 8.9 | 11.80% | 9.8 | 12.50% | 0.4 | 2.00% |
| 1. *Odorrana tormota* | 0.727±0.074 | 2.6 | 1.50% | 19.6 | 15.60% | 1.5 | 12.60% | 16.6 | 15.50% | 0 | 1.80% | 5.4 | 5.80% | 20.3 | 16.80% | 14 | 18.90% | 20 | 11.50% |
| 1. *Pachytriton feii* | 0.925±0.023 | 7.4 | 13.30% | 13.3 | 14.00% | 13.8 | 20.20% | 12.6 | 9.50% | 11.9 | 3.50% | 18.9 | 13.50% | 6.9 | 12.80% | 5.9 | 7.90% | 9.4 | 5.20% |
| 1. *Pelophylax nigromaculatus* | 0.863±0.030 | 0.2 | 0.10% | 3.4 | 0.20% | 0.5 | 0.60% | 35.8 | 44.40% | 0.9 | 0.30% | 3.4 | 4.00% | 0.3 | 0.20% | 6.2 | 18.40% | 49.2 | 31.80% |
| 1. *Polypedates braueri* | 0.846±0.072 | 5.5 | 4.00% | 17.6 | 17.40% | 5.4 | 12.00% | 6 | 2.10% | 0.8 | 2.30% | 44.4 | 33.10% | 13.4 | 19.30% | 5.1 | 6.20% | 1.8 | 3.40% |
| 1. *Quasipaa spinosa* | 0.899±0.028 | 8.3 | 5.80% | 14.9 | 7.20% | 14.4 | 3.70% | 0 | 0.00% | 19.9 | 12.50% | 5.1 | 1.60% | 27 | 7.20% | 5.4 | 53.10% | 5 | 8.90% |
| 1. *Rana zhenhaiensis* | 0.834±0.048 | 9.4 | 10.50% | 12.5 | 5.10% | 24.1 | 7.10% | 43.2 | 39.80% | 0.6 | 3.50% | 1.3 | 1.00% | 1.2 | 0.20% | 1.3 | 30.50% | 6.5 | 2.40% |
| 1. *Staurois wuyiensis* | 0.878±0.036 | 0.5 | 0.30% | 0 | 0.00% | 3.7 | 3.10% | 50.1 | 58.90% | 9.9 | 2.10% | 14.6 | 5.80% | 4.1 | 0.40% | 1.4 | 23.80% | 15.9 | 5.60% |
| 1. *Zhangixalus dennysi* | 0.757±0.059 | 15.3 | 10.00% | 2.7 | 1.60% | 1.3 | 1.40% | 49.4 | 38.70% | 3.1 | 6.30% | 8.5 | 11.20% | 3.6 | 8.30% | 4.9 | 5.20% | 11.1 | 17.30% |

PI: Permutation importance; PC: Percentage contribution.

**Table S2.** List of Amphibian Survey Results in the Mt. Huangshan**.**

| **Famliy** | **Species** | **IUCN(2024)** | **Dominance Index** |
| --- | --- | --- | --- |
| Salamandridae | *Pachytriton feii* | NT | 0.054 |
| Cynops | *Cynops orientalis* | LC | 0.011 |
| Cryptobranchidae | *Andrias davidianus* | CR | 0.004 |
| Bufonidae | *Bufo gargarizans* | LC | 0.040 |
| Megophryidae | *Boulenophrys boettgeri* | VU | 0.071 |
| Hylidae | *Hyla chinensis* | LC | 0.038 |
| Hylidae | *Hyla sanchiangensis* | LC | 0.001 |
| Ranidae | *Rana catesbeianus* | LC | 0.001 |
| Ranidae | *Rana zhenhaiensis* | LC | 0.002 |
| Ranidae | *Amolops wuyiensis* | LC | 0.157 |
| Ranidae | *Nidirana mangveni* | NE | 0.026 |
| Ranidae | *Hylarana latouchii* | LC | 0.016 |
| Ranidae | *Odorrana graminea* | LC | 0.014 |
| Ranidae | *Odorrana tormota* | VU | 0.053 |
| Ranidae | *Odorrana tianmuii* | LC | 0.059 |
| Ranidae | *Pelophylax nigromaculatus* | NT | 0.016 |
| Ranidae | *Fejervarya multistriata* | LC | 0.194 |
| Ranidae | *Limnonectes fujianensis* | NT | 0.004 |
| Ranidae | *Quasipaa spinosa* | VU | 0.081 |
| Rhacophoridaae | *Zhangixalus dennysi* | LC | 0.001 |
| Rhacophoridaae | *Polypedates braueri* | LC | 0.040 |
| Microhylidae | *Microhyla fissipes* | LC | 0.029 |
| Microhylidae | *Microhyla heymonsi* | LC | 0.076 |

Legend: CR: critically endangered; EN: endangered; VU: vulnerable; NT: near threatened; LC: least concern; DD: data deficient; NE: not evaluated;.

**Table S3.** The Mt. Huangshan Amphibian Diversity Index Across Different Months.

| **Month/Index** | **Number** | **Species** | **Shannon** | **Simpson** | **Pielou** | **Chao1** |
| --- | --- | --- | --- | --- | --- | --- |
| 3 | 67 | 7 | 1.618 | 0.529 | 0.576 | 7.5 |
| 4 | 358 | 17 | 3.138 | 0.848 | 0.767 | 19 |
| 5 | 381 | 15 | 3.619 | 0.906 | 0.926 | 15 |
| 6 | 505 | 16 | 3.272 | 0.857 | 0.818 | 16 |
| 7 | 455 | 18 | 3.481 | 0.886 | 0.834 | 18.3 |
| 8 | 176 | 14 | 2.908 | 0.834 | 0.785 | 14.5 |
| 9 | 40 | 7 | 2.611 | 0.817 | 0.920 | 7 |
